# Relationship between fear of progression and symptom burden, disease factors and social/family factors in patients with stage‐IV breast cancer in Shandong, China

**DOI:** 10.1002/cam4.6749

**Published:** 2024-03-08

**Authors:** Qianrun Lu, Qiuyue Liu, Shu Fang, Yujin Ma, Baoxuan Zhang, Huihui Li, Lihua Song

**Affiliations:** ^1^ Department of Breast Cancer Internal Medicine Shandong Cancer Hospital and Institute, Shandong First Medical University and Shandong Academy of Medical Sciences Jinan China

**Keywords:** ECOG, fear of progression, stage‐IV breast cancer, symptom burden

## Abstract

**Objective:**

To assess fear of progression (FoP)'s relationship with symptom burden and disease and social/family factors, as well as, determine the status of FoP in women with stage‐IV breast cancer in Shandong, China.

**Methods:**

Two hundred and sixteen women were recruited from the department of breast cancer internal medicine, Shandong Cancer Hospital and Institute. Data for this observational study were collected between October 2020 and January 2021 using the MD Anderson Symptom Inventory, the Fear of Progression Questionnaire‐Short Form (FoP‐Q‐SF) and a participant information scale. SPSS 23.0 was used for statistical analysis.

**Results:**

After excluding invalid responses, the data of 200 participants were analysed. The average total FoP‐Q‐SF score was 29.39 ± 9.39 (95% confidence interval, 21.81–27.64). The FoP level among the participants was relatively low. For disease and social/family factors, FoP statistically significantly differed by satisfaction with family emotional support and the Eastern Cooperative Oncology Group (ECOG) score. The ECOG score was positively correlated with FoP. Furthermore, symptom burden was positively correlated with FoP.

**Conclusions:**

Among patients with stage‐IV breast cancer, satisfaction with family emotional support, ECOG score and symptom burden play key roles in FoP. Interventions, including providing appropriate emotional support from family, improving physical fitness and relieving symptom burden, must be considered in future studies, which may improve patients' overall physical and mental status and provide a supportive therapeutic environment.

## Graphical abstract

Among patients with stage‐IV breast cancer, satisfaction with family emotional support, ECOG score and symptom burden play key roles in the fear of progression (FoP).

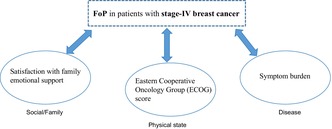

## 1 INTRODUCTION

According to the International Agency for Research on Cancer, breast cancer was the most frequently diagnosed cancer among women in 2020.^1^ The incidence of breast cancer in China is increasing at twice the global rate.^2^ This is alarming because breast cancer is one of the most fatal diseases and greatly impacts patients' psychological, emotional and social well‐being, as well as their family lives.^3^ In clinical work, we find that these influences stem mainly from patients'^4^, ^5^, ^6^, ^7^ and their families'^8^ fear of cancer,^4^ treatment effects,^5^ side effects,^6^ and disease progression.^7^ When considering the unpredictability of the progression of the disease,^9^ fear of disease among people with cancer is far higher than with other chronic diseases such as hypertension and diabetes.

Dankert et al.^10^ presented the concept of fear of progression (FoP), defined as the fear ‘that the illness will progress with all its biopsychosocial consequences or that it will recur’.^11^ Simard et al.^12^ reported that approximately 50% of patients with cancer experienced moderate‐to‐severe FoP. FoP is an adequate response to real threats related to diagnosis, treatment and disease course. The disease or symptom burden can affect patients' FoP status in real time, which means FoP has high clinical relevance.^13^ Further, FoP is negatively correlated with quality of life.^14^

Fear of progression levels differ by cancer type.^15^, ^16^, ^17^ According to Yang et al.,^17^ breast cancer survivors have lower FoP levels than patients with nasopharynx, colorectal and lung cancers. Sun et al.^18^ compared breast, nasopharynx, leukaemia and colorectal cancer survivors and found the same results as Yang et al. However, their patients were mostly in stages II–III. In the only study on Chinese patients with breast cancer,^19^ the 342 participants were divided into four groups by cancer stage; stage‐IV patients had higher FoP than others, suggesting that if we want to deeply understand the FoP of patients with breast cancer, a stage‐wise analysis is required.

An investigation indicated that 55%–90% of patients with breast cancer report fear of cancer recurrence (FCR) throughout survivorship.^20^ Some scholars do not differentiate between FCR and FoP.^21^, ^22^ However, from the oncological perspective, the two are different.^23^ FCR may be applicable to patients with early‐stage cancer, especially those who have undergone surgical resection of the lesion; they may have a sense of fear of the disease before having experienced a relapse. FoP may be applicable to patients with advanced cancer; in this case, they may be living with tumours and worry about the cancer continuing to progress. FoP is more closely related to clinical biological therapy; for example, when a patient's lesions shrink after treatment, their FoP may decrease. Understandably, cancer patients also have death anxiety and fear. Coutts‐Bain et al.^24^ found that in contrast to FCR, death anxiety is more closely associated with FoP. From the standpoint of a couple, the presence of a husband's apprehension towards death or disease progression in cases of advanced cancer was found to be correlated with an increased level of patient symptom burden.^25^ Therefore, it is important to identify factors that can help patients with advanced cancer face their disease and life better.

Aside from the burden of physical symptoms, patients with stage‐IV breast cancer face psychological and social pressures. In three studies on FoP across cancer types, patients with breast cancer accounted for approximately 80% of all patients.^15^, ^17^, ^18^ Patients who were younger,^15^ unemployed^15^, ^17^ and single^15^, ^18^ had received chemotherapy,^15^ a family history of cancer,^17^ a severe childhood illness experience,^15^ depressive/anxiety symptoms^15^, ^17^, ^18^ and low‐income levels^15^, ^17^ tended to report higher FoP. In contrast, patients who were older,^17^ employed full‐time,^17^ had complications,^18^ higher income levels,^15^ and had been diagnosed with breast cancer^17^ tended to report lower FoP. However, the number of patients in stages II–III accounted for 80%–90% of the survey population in the three studies mentioned above. Currently, there is no report on FoP in patients with stage‐IV breast cancer. For patients with advanced breast cancer, especially stage‐IV, the clinical treatment is complicated. As clinicians must consider the patient situation comprehensively when developing treatment plans, a stage‐IV‐specific examination is worthy of our attention.

Therefore, we conducted a questionnaire survey to assess FoP's relationship with symptom burden and disease and social/family factors, as well as determine the status of FoP in patients with stage‐IV breast cancer in Shandong, China. We also sought to identify the relevant factors related to FoP in patients with stage‐IV breast cancer as this would become the basis for oncologists to provide physical and mental treatment that meets individual patients' needs.

## 2 METHODS

### 2.1 Design

This is a cross‐sectional study.

### 2.2 Sample

A total of 216 women with stage‐IV breast cancer were recruited from the Department of Breast Cancer Internal Medicine, Shandong Cancer Hospital and Institute. The participants were all hospitalised. The data were collected between October 2020 and January 2021. Inclusion criteria were: (1) women, (2) histological and imaging confirmation of stage‐IV breast cancer, (3) informed consent and willingness to participate and (4) ability to clearly express one's feelings and thoughts. The exclusion criteria were: (1) unwillingness to participate, (2) stage of breast cancer unknown and (3) communication difficulties or language disorders, such as unclear consciousness caused by brain metastasis, visual impairment and deafness.

### 2.3 Instruments

Three questionnaires were employed to collect data.

#### 2.3.1 Participant information scale

The participant information scale created for this study includes 20 items covering family and social demography, general status and disease factors (Table 1). The family and social demography section includes age, education, occupation, marriage, medicare, satisfaction with family financial support, satisfaction with family emotional support and familial history of tumour. The general status section includes the Eastern Cooperative Oncology Group (ECOG) score and average sleep time within the last 2 weeks. The disease factors section includes current treatment, chemotherapy cycles (except adjuvant therapy), radiotherapy cycles (except adjuvant therapy), endocrine therapy time (except adjuvant therapy), time for breast cancer, Time for IV‐stage, The number of metastatic foci, complications, molecular typing and initial diagnosis. Owing to the technical nature of the disease factors, the researchers went over the relevant content in the submitted questionnaires and ensured that it was concordant with the information obtained from inpatient medical records, including items patients had not clearly answered. The ‘Satisfaction with family emotional support’ factor was used to investigate patients' subjective evaluations of family members' emotional contributions in their daily lives. It was rated on a five‐point Likert scale as follows: (1) very unsatisfied, (2) unsatisfied, (3) neutral, (4) satisfied and (5) very satisfied. Based on the ECOG scoring table,^26^ activity status was divided into six levels. Oncologists commonly use the ECOG score as an indicator of a patient's general health status and tolerance to treatment based on their physical strength. Regarding scoring, 0 indicates ‘fully active, able to carry on all pre‐disease performance without restriction’ and 5 indicates ‘dead’.

**TABLE 1:** Characteristic of patients' disease, family/social factors and analysis of the difference between these factors and FoP (*N* = 200).

Item	*n* (%)	FOP (M ± SD)	Levene test	F/H	*p*
Family and social demography					
Age					
≤30	3 (1.5)	26.33 ± 12.66	0.642	0.561	0.571
31 ~ 49	103 (51.5)	30.01 ± 9.05			
≥50	94 (47.0)	28.81 ± 9.71			
Education					
Primary school and below	44 (22.0)	27.5 ± 11.06	0.065	1.388	0.248
Junior school	68 (34.0)	30.81 ± 9.14			
High/technical secondary school	56 (28.0)	28.57 ± 8.71			
University or higher	32 (16.0)	30.41 ± 8.32			
Occupation					
Full‐time	60 (30.0)	30.77 ± 9.17	0.111	1.25	0.293
Part‐time	4 (2.0)	29 ± 6.78			
Unemployed	101 (50.5)	29.45 ± 10.17			
Retirement	35 (17.5)	26.91 ± 7.20			
Marriage					
Never married	3 (1.5)	28.33 ± 7.37	0.259	0.508	0.603
Currently married	191 (95.5)	29.52 ± 9.50			
Divorce	6 (3.0)	25.67 ± 5.99			
Medicare					
Urban workers	150 (75.0)	29.09 ± 9.27	0.943	0.4	0.671
Urban residents	33 (16.5)	29.85 ± 10.04			
New rural cooperative Medical	17 (8.5)	31.12 ± 9.48			
Satisfaction with family financial support					
Very unsatisfied	4 (2.0)	27.25 ± 9.85	0.332	2.137	0.078
Unsatisfied	3 (1.5)	44.67 ± 15.37			
General	26 (13.0)	29.77 ± 10.49			
Satisfied	76 (38.0)	29.03 ± 8.95			
Very satisfied	91 (45.0)	29.18 ± 8.99			
Satisfaction with family emotional support					
Very unsatisfied	4 (2.0)	25.75 ± 6.13	0.71	3.33	0.012
Unsatisfied	3 (1.5)	42.33 ± 11.02			
General	23 (11.5)	33.65 ± 10.54			
Satisfied	81 (40.5)	28.05 ± 8.73			
Very satisfied	89 (44.5)	29.24 ± 9.24			
Familial history of tumour					
Yes	30 (15.0)	31.2 ± 9.48	0.766	1.314	0.253
No	170 (85.0)	29.07 ± 9.36			
The general status					
ECOG					
0	33 (16.5)	26.39 ± 9.07	0.885	4.43	0.002
1	110 (55.0)	28.45 ± 9.09			
2	44 (22.0)	34.25 ± 8.84			
3	10 (5.0)	29.1 ± 8.8			
4	3 (1.5)	26.33 ± 13.58			
Average sleep time within last two weeks					
≤4 h	8 (4.0)	30.38 ± 10.31	0.74	1.832	0.143
4 h ~ 6 h	47 (23.5)	32.09 ± 9.06			
>6 h ~ 8 h	103 (51.5)	28.57 ± 9.05			
>8 h	42 (21.0)	28.19 ± 10.10			
Disease factors					
Current treatment					
Including chemotherapy	137 (68.5)	30.22 ± 9.59	0.797	1.204	0.306
Radiotherapy	5 (2.5)	24 ± 8.69			
Endocrine + targeted	6 (3.0)	25.83 ± 9.68			
Endocrine	31 (15.5)	28.48 ± 9.17			
Targeted	8 (4.0)	31.38 ± 8.91			
Immunotherapy	2 (1.0)	21 ± 5.66			
No treatment	11 (5.5)	26.09 ± 7.15			
Chemotherapy cycles (except adjuvant therapy)					
0	42 (21.0)	29.74 ± 8.02	0.045	2.518	0.641
1 ~ 4	50 (25.0)	27.92 ± 7.69			
5 ~ 8	59 (29.5)	29.2 ± 10.3			
9 ~ 12	22 (11.0)	31.82 ± 9.86			
13 or more	27 (13.5)	30 ± 11.68			
Radiotherapy cycles (except adjuvant therapy)					
0	128 (64.0)	28.65 ± 9.15	0.928	0.917	0.455
1	31 (15.5)	31.32 ± 9.06			
2	9 (4.5)	27.89 ± 10.66			
3	4 (2.0)	34 ± 10.1			
4 or more	28 (14.0)	30.46 ± 10.38			
Endocrine therapy time (except adjuvant therapy) (*N* = 133)					
No	48 (36.1)	28.46 ± 9.34	0.394	0.855	0.513
≤3 m	18 (13.5)	32.32 ± 10.04			
3 m ~ 6 m	9 (6.8)	31.6 ± 11.05			
>6 m ~ 12 m	17 (12.8)	28.43 ± 7.94			
>1 y ~ 2 y	18 (13.5)	30.67 ± 8.98			
>2 y	23 (17.3)	29.65 ± 9.82			
Time for breast cancer					
≤3 y	85 (42.5)	28.79 ± 9.44	0.892	0.305	0.738
>3 y ~ 5 y	42 (21.0)	29.93 ± 9.43			
>5 y	73 (36.5)	29.78 ± 9.39			
Time for IV‐stage					
≤6 m	62 (31.0)	30.11 ± 8.81	0.563	0.607	0.546
>6 m ~ 2 y	79 (39.5)	28.49 ± 9.30			
>2 y	59 (29.5)	29.83 ± 10.12			
The number of metastatic foci					
1	59 (29.5)	28.61 ± 8.73	0.05	0.528	0.715
2	67 (33.50)	29.81 ± 9.55			
3	47 (23.5)	29.13 ± 8.04			
4	24 (12.0)	31.21 ± 12.33			
5	3 (1.5)	25 ± 14.93			
Complications					
Yes	47 (23.5)	28.83 ± 8.33	0.144	0.218	0.641
No	153 (76.5)	29.56 ± 9.71			
Molecular typing					
Luminal A	49 (24.5)	29.69 ± 9.32	0.842	0.458	0.766
Luminal B (HER2‐)	50 (25.0)	28.22 ± 10.06			
Luminal B (HER2+)	34 (17.0)	31 ± 10.11			
HER‐2 overexpression	35 (17.5)	29.29 ± 8.49			
Triple negative	32 (16.0)	29.16 ± 8.83			
Initial diagnosis					
I–III	160 (80.0)	29.62 ± 9.29	0.289	0.474	0.492
IV	40 (20.0)	28.48 ± 9.84			

*Note*: ECOG, Eastern Cooperative Oncology Group; *p < 0.05, **p < 0.01.

#### 2.3.2 Fear of Progression Questionnaire‐Short Form

The Fear of Progression Questionnaire‐Short Form (FoP‐Q‐SF) was used to measure FoP. It has been validated for use on people with breast cancer.^27^ The instrument contains 12 items across the physiological health and social/family dimensions (Table 2). The items are scored on a five‐point Likert scale from 1 (never) to 5 (very often). The total score ranges from 12 to 60; the higher the score, the higher the level of FoP. A total score ≥34 is indicative of mental disorders.^28^

**TABLE 2:** FoP‐Q‐SF statistical description (*N* = 200).

Items	Score (mean ± SD)	Parameter estimate (95% CI)
10. Worrying that medications could damage the body	2.87 ± 1.14	2.71–3.02
2. Being nervous prior to doctors' appointments or periodic examinations	2.81 ± 1.20	2.64–2.98
11. Worrying about what will happen to the family if something should happen to the patient	2.80 ± 1.14	2.64–2.96
3. Being afraid of pain	2.70 ± 1.13	2.54–2.85
1. Being afraid of disease progression	2.67 ± 0.93	2.54–2.79
9. Being afraid of severe medical treatments in the course of the illness	2.64 ± 1.06	2.49–2.79
4. Being afraid of becoming less productive at work	2.41 ± 1.17	2.25–2.57
12. Being afraid of not being able to work anymore	2.35 ± 1.24	2.17–2.52
5. Having physical symptoms (e.g. rapid heartbeat, stomach ache, nervousness)	2.31 ± 0.96	2.18–2.44
8. Fear of no longer being able to pursue hobbies	2.09 ± 1.05	1.94–2.23
6. Being afraid of the possibility that the children could contract cancer	2.04 ± 1.15	1.88–2.20
7. Being afraid of relying on strangers for activities of daily living	1.73 ± 0.96	1.59–1.86
Physiological health dimension (Items 1, 2, 3, 5, 9, 10)	15.99 ± 4.82	11.11–14.04
Social/family dimension (Items 4, 6, 7, 8, 11, 12)	13.41 ± 5.03	10.60–13.70
Total score	29.39 ± 9.39	21.81–27.64

*Note*: The items are ordered by score.

Abbreviations: CI, confidence interval; FoP‐Q‐SF, Fear of Progression Questionnaire‐Short Form.

#### 2.3.3 MD Anderson Symptom Inventory

The MD Anderson Symptom Inventory (MDASI) was used to measure symptom burden. The questionnaire includes two subscales: symptom severity and symptom interference (Table 3). The symptom severity scale comprises 13 items (fatigue (tiredness), problem with remembering things, distressed (upset), disturbed sleep, lack of appetite, dry mouth, sadness, Pain, numbness or tingling, drowsy (sleepy), nausea, shortness of breath and vomiting) that correspond to the core symptoms used in the general MDASI and 6 items (breast changes, fingernail or toenail changes, skin changes, hot flashes, arm swelling, constipation) that are common symptoms related to breast cancer or treatment, according to the MDASI‐Breast.^29^ The symptom interference scale consists of six items that measure a person's perceived interference of the symptoms in different aspects of daily life (Table 3). Symptoms were rated ‘at their worst’ in the previous 24 h on a 10‐point numeric scale ranging from 0 (not present) to 10 (as bad as you can imagine); these were subsequently categorised as mild,^1^, ^2^, ^3^, ^4^ moderate,^5^, ^6^ or severe.^7^, ^8^, ^9^, ^10^ The interference subscale items were rated similarly, with 0 representing ‘did not interfere’ and 10 ‘interfered completely’.

**TABLE 3:** MD Anderson Symptom Inventory statistical description and correlation analysis (*N* = 200).

Items	Mean ± SD	Parameter estimate (95% CI)	Moderate and Severe (%)	None *n* (%)	*r*/rs	*p*
Symptom severity score	2.02 ± 1.62	1.80–2.25			0.522	<0.001
Fatigue (tiredness)	2.76 ± 2.32	2.44–3.08	52 (26.0)	38 (19.0)	0.320	<0.001
Breast changes	2.50 ± 2.90	2.10–2.90	47 (23.5)	70 (35.0)	0.529	<0.001
Problem with remembering things	2.43 ± 2.35	2.10–2.76	39 (19.5)	49 (24.5)	0.322	<0.001
Distressed (upset)	2.31 ± 2.67	1.94–2.68	49 (24.5)	76 (38.0)	0.505	<0.001
Disturbed sleep	2.30 ± 2.50	1.95–2.65	39 (19.5)	70 (35.0)	0.302	<0.001
Lack of appetite	2.23 ± 2.59	1.86–2.59	41 (20.5)	79 (39.5)	0.336	<0.001
Dry mouth	2.20 ± 2.34	1.87–2.52	34 (17.0)	60 (30.0)	0.334	<0.001
Fingernail or toenail changes	2.13 ± 2.65	1.76–2.49	40 (20.0)	83 (41.5)	0.245	<0.001
Sadness	2.07 ± 2.73	1.68–2.45	41 (20.5)	93 (46.5)	0.474	<0.001
Skin changes	1.98 ± 2.33	1.65–2.30	33 (16.5)	81 (40.5)	0.334	<0.001
Pain	1.91 ± 2.38	1.57–2.24	31 (15.5)	78 (39.0)	0.241	0.001
Numbness or tingling	1.91 ± 2.70	1.53–2.29	38 (19.0)	102 (51.0)	0.312	<0.001
Hot flashes	1.80 ± 2.26	1.48–2.11	27 (13.5)	87 (43.5)	0.321	<0.001
Drowsy (sleepy)	1.76 ± 2.17	1.46–2.06	30 (15.0)	89 (44.5)	0.290	<0.001
Arm swelling	1.74 ± 2.46	1.39–2.08	31 (15.5)	97 (48.5)	0.298	<0.001
Constipation	1.70 ± 2.45	1.35–2.04	30 (15.0)	100 (50.0)	0.310	<0.001
Nausea	1.65 ± 2.54	1.29–2.00	32 (16.0)	108 (54.0)	0.269	<0.001
Shortness of breath	1.65 ± 2.16	1.34–1.95	26 (13.0)	92 (46.0)	0.355	<0.001
Vomiting	1.49 ± 2.57	1.13–1.84	26 (13.0)	125 (62.5)	0.309	<0.001
Symptom interference score	1.99 ± 2.05	1.70–2.27			0.496	<0.001
Work (including housework)	2.64 ± 3.00	2.22–3.05	52 (26.0)	68 (34.0)	0.376	<0.001
Mood	2.11 ± 2.33	1.78–2.43	33 (16.5)	66 (33.0)	0.536	<0.001
General activity	1.94 ± 2.58	1.58–2.30	37 (18.5)	96 (48.0)	0.299	<0.001
Walking	1.87 ± 2.56	1.51–2.23	33 (16.5)	101 (50.5)	0.237	0.001
Enjoyment of life	1.85 ± 2.50	1.50–2.19	36 (18.0)	99 (49.5)	0.520	<0.001
Relations with others	1.53 ± 2.42	1.19–1.87	34 (17.0)	119 (59.5)	0.438	<0.001

*Note*: The items are ordered by score.

### 2.4 Data collection

The electronic ‘Questionnaire Star’ tool (https://www.wjx.cn/) was used to administer the questionnaires. First, we obtained permission from the patients' doctors. Second, individuals who met the inclusion criteria were invited to participate in the survey, and the study's purpose, significance and confidentiality were explained. Third, the patients were instructed to scan the QR code with their phone (WeChat) or type the URL directly into the browser. We collected 216 questionnaires. Questionnaires were considered invalid if: (1) there were unexpected interruptions, such as patients feeling unwell or being unwilling to continue; (2) important information was missing; and (3) there were repeated questionnaires for the same patient, in which case we used only the first.

### 2.5 Statistical analysis

The data were analysed using SPSS 23.0 (IBM Corp., Armonk, NY, USA). The participant information scale consisted of grade data and counting data, described using frequency and percentage. The scores of the MDASI and FoP‐Q‐SF constituted measurement data, described using mean and standard deviation. Shapiro–Wilk normality analysis was used to test the normality of the distribution of FoP‐Q‐SF scores based on the factors among groups. If the distribution was normal, Levene's test for homogeneity of variance was used. If the variance was homogeneous, single‐factor analysis of variance was performed to analyse the differences in FoP‐Q‐SF scores among groups. The Kruskal–Wallis test was used if the data did not comply with the parametric assumption of normality and homogeneity of variance. The data correlation between factors (the participant information scale) and FoP‐Q‐SF scores were analysed using Spearman's analysis. Factors (the participant information scale) with *p* < 0.05 in the single‐factor analysis of variance were analysed using Spearman's correlation analysis. The correlation between symptom burden and FoP was analysed using Pearson's correlation coefficient. Statistical significance was defined as *p* < 0.05 (two‐tailed). Cronbach alpha coefficient was used to test the reliability of FoP‐Q‐SF.

### 2.6 Ethical considerations

Ethical approval was obtained from the Institutional Review Board of Shandong Cancer Hospital and Institute (SDTHEC2020009010). All participants provided informed consent.

## 3 RESULTS

### 3.1 Sample characteristics

Of the 216 participants included in this study, 25 (12%) accessed the questionnaire via URL, while 191 (88%) used the QR code. After deleting invalid questionnaires (please refer to the Data Collection section for criteria), 200 were used in the analysis (effective response rate: 92.59%). Of these 200 questionnaires, 24 (12%) were accessed via URL, while 176 (88%) were accessed via QR code. The participants' age range was 20–75 years (mean: 49.47; median: 50) (Table 1). The time since breast cancer diagnosis ranged from 0 to 255 months (mean: 60.92; median: 41.50). The time since stage‐IV breast cancer diagnosis ranged from 0 to 150 months (mean: 20.24; median: 12.50). It was determined that 189 (94.5%) participants were receiving anti‐tumour therapy, and 141 (70.5%) had two or more metastatic lesions.

### 3.2 FoP‐Q‐SF

The average total score of the FoP‐Q‐SF was 29.39 ± 9.39 (95% confidence interval, 21.81–27.64) (Table 2). The score revealed that 72 (36%) individuals had mental dysfunction. The three highest scoring items were ‘Worrying that medications could damage the body’, ‘Being nervous prior to doctors’ appointments or periodic examinations' and ‘Worrying about what will happen to the family if something should happen to the patient’. The instrument had good reliability, with a Cronbach's *α* of 0.84 for physiological health and 0.84 for social/family factors.

### 3.3 Participant information scale and univariate analysis

Fear of progression differed significantly by satisfaction with family emotional support and ECOG score, suggesting the relevance of physical status and family emotional support to FoP in patients with stage‐IV breast cancer. Spearman's correlation analysis showed a positive correlation between ECOG score (rs = 0.230, *p* = 0.002) and FoP.

### 3.4 MDASI and correlation analysis

Pearson's correlation analysis suggested that all MDASI items were statistically significantly positively correlated with FoP. A positive correlation exists between the symptom severity score (*r* = 0.522, *p* < 0.001) and FoP. A positive correlation exists between the symptom interference score (*r* = 0.496, *p* < 0.001) and FoP. There were 134 (67%) patients with moderate and severe symptom severity and 77 (38.5%) patients with moderate and severe symptom interference (Table 3). The symptom burden in stage‐IV breast cancer was severe.

## 4 DISCUSSION

We sought to analyse FoP's relationship with symptom burden and disease and social/family factors as well as determine the status of FoP in patients with stage‐IV breast cancer. The results showed that the FoP among women with stage‐IV breast cancer was relatively low. Regarding factors, FoP varied by satisfaction with family emotional support and ECOG. The ECOG and symptom burden both displayed positive associations with FoP.

In 2019, in Guangdong, China, Yang et al.^17^ administered the FoP‐Q‐SF to 1025 patients with cancer. Nearly 80% of the patients had been diagnosed with breast cancer; they tended to have lower FoP than patients with other types of cancer. The next year, Chung et al.^30^ completed an observational study on FoP in 311 patients with cancer in Hong Kong; of these, about 28% had breast cancer. It is generally believed that patients with more severe disease have a higher FoP. In the above two pan‐cancer studies in China, breast cancer accounted for the highest proportion, but stage‐IV accounted for the least. However, the FoP score in our study was similar to theirs. Therefore, compared with all types of cancer, FoP associated with stage‐IV breast cancer was lower. This may be because of two reasons. First is the cultural background. Shandong is strongly influenced by Confucian ideology, such as a humble and stable attitude towards life. When facing difficulties, the people of Shandong may display more calmness and less fear or anxiety than people from other regions. Second is the medical background. We are their doctors. Differently, in clinical work, we particularly pay attention to the health and disease education of patients, through face to face (in hospital) and network (at home) communication, to ensure that patients can receive treatment and live with peace of mind.

Fear of progression has been reported in numerous studies.^13^, ^15^, ^16^, ^17^, ^18^, ^19^, ^30^, ^31^, ^32^ However, these have generally focused on early‐stage cancer or pan‐cancer/pan‐stage examinations. There is limited data regarding stage‐IV breast cancer. In Niu et al.'s^19^ survey of 342 Chinese stage I–IV breast cancer patients (stage‐IV cancer patients accounted for only 24.9% of the sample), the average FoP score was significantly higher (38.79 ± 5.78) than in our study. We speculate that the difference in results is primarily owing to the disease stage. Patients with early‐stage breast cancer have limited knowledge of the disease and treatment. Therefore, their fear might stem mainly from misperceptions; they are likely to believe that breast cancer is a terminal disease and that progression implies death. Comparing the three items with the highest scores in FoP‐Q‐SF from our results with the above survey, two were the same: ‘Worrying that measurements could damage the body’ and ‘Being nervous before doctors' appointments or periodic examinations’. The item with a difference between our study and the one by Niu et al. was ‘Being afraid of disease progression’, which confirms our conjecture above. Our study was ‘Worrying about what will happen to the family if something should happen to the patient’. Patients with stage‐IV breast cancer experience longer treatment and more spiritual pain than those in the early stages. It is possible that by this stage, they have accepted their diagnosis and focus more on their family. People may experience posttraumatic growth^33^ after experiencing major tribulations and become more resilient.^34^ Posttraumatic growth refers to a positive response to stressful events. Resilience refers to facing difficulties and setbacks with a better attitude. Patients with stage‐IV breast cancer may have experienced personal growth after the initial stress of the diagnosis. This could account for their relatively calm outlook on life and lower FoP.

We discovered that FoP statistically significantly differed by satisfaction with family emotional support and ECOG scores. A higher ECOG score corresponded to higher FoP. During clinical treatment, most patients with stage‐IV breast cancer experience many instances of disease progression and few effective treatments. Furthermore, in the Chinese Confucian context, women with breast cancer suffer alone because they are afraid of burdening their families.^35^ However, they long for emotional support. Social support is a broad construct that describes an individual's perceived network of social resources.^36^ It includes subjective and objective support. Objective support refers to visible or practical support, whereas subjective support refers to the individual's perceptions of society's respect and understanding.^37^ Family emotional support falls under subjective support. When a patient feels cared for by family, friends, doctors and nurses, their FoP is reduced, and stress is effectively absorbed.^37^, ^38^ Therefore, emotional support from the family is particularly important in the context of stage‐IV breast cancer.

We found that the ECOG score was also positively correlated with FoP. The worse the patient's physical condition, the higher their FoP. It is simple and feasible in clinical work to identify patients with severe FoP by paying attention to their physical condition, which has significant guiding value for clinical oncology medical workers.

We summarised common symptoms among patients with breast cancer and found that they were positively correlated with FoP. From the perspective of clinical oncologists, this is consistent with the actual situation; patients' disease condition and symptom burden do significantly affect their psychological well‐being. If the disease or symptom burden make patients uncomfortable, they will show fear and concern about cancer. A previous study revealed a significant positive correlation between symptom burden and FoP.^39^ In a study on Chinese American patients with breast cancer in stages I–III, pain and fatigue were associated with higher FCR.^40^ These results are consistent with our findings. In our sample, 94.5% of the patients were in treatment, and their symptom burden was relatively heavy. Fatigue was the most severe symptom, followed by ‘breast changes’ and ‘problem with remembering’. A survey on symptom burden among Israeli women with breast cancer using the MDASI questionnaire revealed that fatigue was the most common and severe symptom.^41^ A set of common cancer‐ and treatment‐related symptoms were investigated in 3106 patients in the United States. The results revealed that, at initial assessment, fatigue was the most common symptom, experienced at a moderate‐to‐severe level.^42^ A 2013 ECOG survey of 1544 patients with breast cancer in the United States revealed that fatigue ranked first.^43^ A literature review of 21 multi‐national studies involving 4067 people with cancer concluded that fatigue is the most common and serious symptom among those receiving active cancer treatment.^44^ Thus, it appears that the impact of symptom burden on FoP is not influenced by cultural differences. Fatigue is the main symptom across all types of cancer.

### 4.1 Limitations

First, given that we only investigated one centre, there may have been selection bias in the survey, as the sample did not include all stage‐IV breast cancer patients in Shandong Province. However, the centre investigated is located at the largest cancer hospital in Shandong Province, the patients of which come from all regions of the province. In addition, we applied the enrollment criteria uniformly and recruited individuals with diverse conditions. This helped ensure the samples' representativeness. Regarding the data collection methods, using a QR code and URL to access the questionnaire did not result in selection bias, even when considering the participants' age or education levels. To the contrary, during the pandemic period, all public places in China were assigned a unique QR code, called ‘place codes’. Accessing these public places was not possible without the place codes. Therefore, Chinese people were already familiar with QR codes at the time of the study. Further, QR codes are common in the hospital wherein this study was conducted, and the vast majority of patients can use them correctly. Moreover, we found that using the URL and QR code was more convenient than using paper questionnaires, as the font size in electronic questionnaires can be adjusted, making it more user‐friendly for older patients. Of course, we remained on standby to assist participants if they expressed difficulties in using QR codes or URLs or in reading the text.

Second, the questionnaires may have caused information bias due to the possibility of everyone having a slightly different understanding of the items. At present, the validation of the FoP‐Q‐SF is mainly based on the type of cancer disease, to the best of our knowledge. In 2006, it was validated for use on patients with breast cancer (including stages I–IV).^27^ Our study is the first to analyse stage‐IV breast cancer patients individually. In our study, the FoP‐Q‐SF had good reliability, with a Cronbach's *α* of 0.84 for physiological health and 0.84 for social/family. In addition, using the Participant Information Scale, we measured the degree of satisfaction with family emotional support on a five‐point Likert scale. Perhaps, a comprehensive questionnaire, such as Spousal Support Perception Scale for Breast Cancer Patients,^45^ will be more effective. However, considering that patients need rest during hospitalisation and our aim was to understand patients' feelings from their perspective, we considered the five‐point Likert scale adequate. Further, we used patient and medical records for the information source of the scale. After the patients completed the questionnaire, we supplemented and corrected their information with and based on their medical records. This avoided information bias to the greatest extent.

Third, we did not adequately explore the overlap between FoP and symptom burden. Symptom burden is a multidimensional concept comprising disease‐related symptoms, directly or indirectly caused by cancer and treatment‐related symptoms, divided into physical and mental pain. While FoP may actually fall under symptom burden, we considered only its clinical characteristics. This may make the conclusions noncomprehensive.

### 4.2 Clinical implications

This study, conducted by clinical oncologists, is not only rare, but also has important clinical implications. First, although FoP is a subject of study in both psychology and oncology fields, patients with cancer might be more willing to talk to doctors (i.e. oncologists) about their feelings than to nurses or clinical psychologists, because such communication is closely related to clinical antitumor therapy. Second, this study allows us to gain a faster understanding of patients' physical and psychological status. It has indeed helped doctors discover previously undetected problems in some patients, such as those associated with pain and sleep and even chemotherapy drug doses. This can help determine the most appropriate course of treatment. Third, our inspiration for the studied factors comes from clinical work. Our results are convincing to oncologists.

This is the first study to explore the status of FoP in patients with stage‐IV breast cancer and its relationship with social demography, disease factors and symptom burden. As the Chinese are generally introverted and not good at expressing their thoughts, the results suggest that oncologists should pay more attention to patients with high ECOG scores or heavy symptom burden, while also emphasising family care. Doctors should patiently communicate with patients to identify the cause of FoP and then try to help them by explaining treatment, solutions to current problems and successful treatment cases, to enhance their confidence in treatment and encourage them to learn from the successful experiences of rehabilitated patients. When patients realise the effect of good physical and mental status for cancer stability and recovery, they may naturally strive to live a positive life. Doctors could also communicate with patients' spouses and families to make them aware of the importance of their behaviours towards patients. Maybe, this way, patients' satisfaction with family emotional support will improve.

### 4.3 Conclusion

We attempted to address key knowledge gaps regarding the status of FoP in patients with stage‐IV breast cancer and its relationship with social demography, disease factors and symptom burden. ECOG score and symptom burden were associated with increased FoP levels. Patients' satisfaction with family emotional support, ECOG scores and symptom burden play key roles in FoP. Interventions, including improving emotional support from family and relieving symptom burden, must be considered in future studies of FoP among women with stage‐IV breast cancer, which may positively affect patients' overall physical and mental status and provide a supportive therapeutic environment.

## AUTHOR CONTRIBUTIONS

**Qianrun Lu:** Conceptualization (lead); data curation (lead); formal analysis (lead); investigation (lead); methodology (lead); project administration (lead); validation (lead); writing – original draft (lead); writing – review and editing (lead). **Qiuyue Liu:** Investigation (lead). **Shu Fang:** Resources (lead). **Yujin Ma:** Investigation (equal). **Baoxuan Zhang:** Resources (lead). **Huihui Li:** Resources (lead). **Lihua Song:** Conceptualization (lead); formal analysis (lead); funding acquisition (lead); investigation (lead); methodology (lead); project administration (lead); resources (lead); writing – review and editing (lead).

## FUNDING INFORMATION

This research did not receive any specific grant from funding agencies in the public, commercial or not‐for‐profit sectors.

## CONFLICT OF INTEREST STATEMENT

None.

## ETHICS STATEMENT

Ethical approval was obtained from the Institutional Review Board of Shandong Cancer Hospital and Institute (SDTHEC2020009010).

## Data Availability

The data are available from the authors on request.
